# Supplementary material for: Placental Characteristics of a Large Italian Cohort of SARS-CoV-2-Positive Pregnant Women
Source: Microorganisms. 2022 Jul 15;10(7):1435. doi: 10.3390/microorganisms10071435 (PMC9317507; doi:10.3390/microorganisms10071435)
Supplement: Supplementary file 1 [file microorganisms-10-01435-s001.zip › microorganisms-1798231-supplementary.pdf]

Table S1. Macroscopic characteristics and maturity of the placentas.

Maternal characteristic	N=975	
	**n**	**%**
Placenta sending procedure (21 missing)		
Stored fresh	54	5.7
Vacuum-packed	3	0.3
Fixed in 10% formalin	897	94.0
Placenta received (15 missing)		
Intact	877	91.4
Fragmented	66	6.9
Torn	17	1.8
Placenta (27 missing)		
Single	935	98.6
Twins	11	1.2
Twins	2	0.2
Placental weight		
<10th centile	91	9.3
10th-90th centile	649	66.6
>90th centile	45	4.6
Missing	190	19.5
Cord attached (7 missing)		
Yes	954	98.6
No	14	1.4
Cord insertion		
Central	615	63.1
Marginal	276	28.3
Velamentous	10	1.0
Missing	74	7.6
Strictures (21 missing)	20	2.1
Knots (21 missing)	57	6.0
Twists (21 missing)	10	1.0
Cord anomalies		
Yes	3	0.3
No	582	59.7
Missing	390	40.0
Membranes		
Complete	240	24.6
Incomplete	186	19.1
Thin	431	44.2
Thickened	52	5.3
Missing	66	6.8
Membrane appearance (27 missing)		
Opaque	202	21.3
Clear	740	78.1
Other	6	0.6
Maturity (29 missing)		

Immature	77	8.1
Consistent with gestational age	686	72.5
Hypermature	48	5.1
Dysmature	135	14.3

Table S2. Placentas by severity of chorioamnionitis and severity of placental disc inflammation (N=975).

Chorioamnionitis	Placental disc inflammation *							
	Absent		Mild		Moderate/severe		Total	
	**n**	**%**	**n**	**%**	**n**	**%**	**n**	**%**
Absent	287	78.6	162	51.4	132	44.7	581	59.6
Mild	38	10.4	89	28.3	96	32.5	223	22.9
Moderate/severe	40	11.0	64	20.3	67	22.7	171	17.5
Total	365	100.0	315	100.0	295	100.0	975	100.0

* Absent inflammation: no intervillous fibrin, no acute, chronic or mixed villitis and intervillitis;
Mild inflammation: mild intervillous fibrin and/or mild acute, chronic or mixed villitis and/or intervillitis;
Moderate or severe inflammation: moderate or severe intervillous fibrin and/or moderate or severe acute, chronic or mixed villitis and/or intervillitis; the inflammation was classified as severe when all three conditions were present.

Table S3. Placentas by severity of funisitis and severity of placental disc inflammation (N=975).

**Funisitis**	**Placental disc inflammation ***							
	**Absent**		**Mild**		**Moderate/severe**		**Total**	
	**n**	**%**	**n**	**%**	**n**	**%**	**n**	**%**
Absent	327	89.6	238	75.6	233	79.0	798	81.8
Mild	29	7.9	57	18.1	51	17.3	137	14.1
Moderate/severe	9	2.5	20	6.3	11	3.7	40	4.1
Total	365	100.0	315	100.0	295	100.0	975	100.0

* Absent inflammation: no intervillous fibrin, no acute, chronic or mixed villitis and intervillitis;
Mild inflammation: mild intervillous fibrin and/or mild acute, chronic or mixed villitis and/or intervillitis;
Moderate or severe inflammation: moderate or severe intervillous fibrin and/or moderate or severe acute, chronic or mixed villitis and/or intervillitis; the inflammation was classified as severe when all three conditions were present.

Table S4. Placentas by severity of chorioamnionitis and presence or absence of COVID-19 pneumonia (N=975).

**Chorioamnionitis**	**COVID-19 pneumonia**					
	**No**		**Yes**		**Total**	
	**n**	**%**	**n**	**%**	**n**	**%**
Absent	533	59.3	48	63.2	581	59.6
Mild	202	22.5	21	27.6	223	22.9
Moderate/severe	164	18.2	7	9.2	171	17.5
Total	899	100.0	76	100.0	975	100.0

COVID-19: Coronavirus Disease 2019.

Table S5. Placentas by severity of funisitis and presence or absence of COVID-19 pneumonia (N=975).

**Funisitis**	**COVID-19 pneumonia**					
	**No**		**Yes**		**Total**	
	**n**	**%**	**n**	**%**	**n**	**%**
Absent	732	81.4	66	86.8	798	81.8
Mild	128	14.2	9	11.8	137	14.1
Moderate/severe	39	4.3	1	1.3	40	4.1
Total	899	100.0	76	100.0	975	100.0

COVID-19: Coronavirus Disease 2019.

Table S6. Placentas by severity of chorionic disc inflammation and presence or absence of COVID-19 pneumonia (N=975).

Chorionic disc inflammation	COVID-19 pneumonia					
	No		Yes		Total	
	n	%	n	%	n	%
Absent	337	37.5	28	36.8	365	37.4
Mild	293	32.6	22	28.9	315	32.3
Moderate/severe	269	29.9	26	34.2	295	30.3
Total	899	100.0	76	100.0	975	100.0

COVID-19: Coronavirus Disease 2019.

Table S7. Placentas by severity of intervillous fibrin deposition and presence or absence of COVID-19 pneumonia (N=975).

Intervillous fibrin deposition	COVID-19 pneumonia					
	No		Yes		Total	
	n	%	n	%	n	%
Absent	364	40.5	29	38.2	393	40.3
Mild	336	37.4	25	32.9	361	37.0
Moderate/severe	167	18.6	20	26.3	187	19.2
Total	32	3.6	2	2.6	34	3.5

COVID-19: Coronavirus Disease 2019.

Table S8. Types of inflammatory infiltrate by presence or absence of COVID-19 pneumonia (N=975).

**Inflammatory infiltrate**	**COVID-19 pneumonia**					
	**No**		**Yes**		**Total**	
	**n**	**%**	**n**	**%**	**n**	**%**
Absent	758	84.3	63	82.9	821	84.2
Lymphoplasmacytic	2	0.2	0	0.0	2	0.2
Lymphogranulocytic	50	5.6	6	7.9	56	5.7
Lymphohistiocityc	89	9.9	7	9.2	96	9.8
Total	899	100.0	76	100.0	975	100.0

COVID-19: Coronavirus Disease 2019.

Table S9. Placentas by presence/absence of intervillar haemorrages and severity of placental inflammation (N=975).

Intervillar haemorrhage	Placental inflammation *							
	Absent		Mild		Moderate/severe		Total	
	n	%	n	%	n	%	n	%
No	291	79.7	201	63.8	172	58.3	664	68.1
Yes	74	20.3	114	36.2	123	41.7	311	31.9
Recent	*23*	*6.3*	*68*	*21.6*	*60*	*20.3*	*151*	*15.5*
In organization	*33*	*9.0*	*36*	*11.4*	*46*	*15.6*	*115*	*11.8*
Organized	*18*	*4.9*	*10*	*3.2*	*14*	*4.7*	*42*	*4.3*
Pseudocistic	*0*	*0.0*	*0*	*0.0*	*3*	*1.0*	*3*	*0.3*
Total	365	100.0	315	100.0	295	100.0	975	100.0

* Absent inflammation: no intervillous fibrin, no acute, chronic or mixed villitis and intervillitis;
Mild inflammation: mild intervillous fibrin and/or mild acute, chronic or mixed villitis and/or intervillitis;
Moderate or severe inflammation: moderate or severe intervillous fibrin and/or moderate or severe acute, chronic or mixed villitis and/or intervillitis; the inflammation was classified as severe when all three conditions were present.

Tables S10. Placentas by presence or absence of infarcts in maternal vascular malperfusion (N=975).

Placental infarcts in MVM	n	%
No	783	80.3
Yes	192	19.7
Recent	*65*	*6.7*
In organization	*64*	*6.6*
Old	*63*	*6.5*
Total	975	100.0

MVM: maternal vascular malperfusion.
