# Supplementary material for: Placental Characteristics of a Large Italian Cohort of SARS-CoV-2-Positive Pregnant Women
Source: Microorganisms. 2022 Jul 15;10(7):1435. doi: 10.3390/microorganisms10071435 (PMC9317507; doi:10.3390/microorganisms10071435)
Supplement: Supplementary file 1 [file microorganisms-10-01435-s001.zip › Supplementary File S1.pdf]

Supplementary File S1. Maternity units in the participating Regions

Piedmont Region

Elena Amoruso *Ospedale Sant'Andrea Vercelli;* Alberto Arnulfo, Enrico Finale *Stabilimento Ospedaliero Castelli Verbania;* Rossella Attini, Marisa Biasio, Luca Marozio, Clara Monzeglio OIRM *Sant'Anna - AOU Città della Salute e della Scienza di Torino;* Maria Bertolino, Andrea Guala *Ospedale San Biagio Domodossola;* Silvia Bonassisa, Alberto De Pedrini *Ospedale Maggiore della Carità Novara;* Mario Canesi, Sara Cantoira *Ospedale Maria Vittoria Torino;* Paola Capelli *Istituto SS. Trinità Borgomanero;* Ilaria Careri, *Ospedale Martini Torino;* Luigi Carratta *Ospedale S. Spirito Casale Monferrato;* Ilaria Costaggini *Ospedale degli Infermi Rivoli;* Tania Cunzolo *Presidio Osp. Cardinal G. MASSAIA Asti;* Enza De Fabiani, Andrea Villasco *Azienda Ospedaliera Ordine Mauriziano Torino;* Cinzia Diano *Ospedale Maggiore Chieri;* Fiorenza Droghini, Paola Rota *Ospedale Santa Croce Moncalieri;* Daniela Kozel, Vittorio Aguggia *Ospedale Civile SS. Antonio e Biagio Alessandria;* Francesca Maraucci *Ospedale degli infermi Biella;* Gisella Martinotti *Ospedale SS. Pietro e Paolo Borgosesia;* Maria Milano, Antonia Novelli *Ospedale Civile Mondovì;* Giovanna Oggè *Ospedale maggiore SS. Annunziata Savigliano;* Simona Pelissetto *Ospedale Civile di Ivrea;* Pasqualina Russo *Presidio Osp. riunito Cirié;* Manuela Scatà *Ospedale Michele e Pietro Ferrero di Verduno;* Federico Tuo, Valentina Casagrande *Ospedale San Giacomo Novi Ligure/Tortona;* Concetta Vardè *Ospedale Agnelli Pinerolo;* Elena Vasario *Azienda Ospedaliera S. Croce e Carle Cuneo*; Daniela Ventrella *Ospedale Civico Chivasso*

Liguria Region

Silvia Andrietti *ASL1 Imperiese*; Federica Baldi *Ospedale San Paolo Savona*; Angelo Cagnacci, Federica Laraud *IRCCS AOU San Martino*; Franco Camandona, Domenico Grimaldi *Ospedale Galliera di Genova*; Maria Franca Corona, Massimiliano Leoni *Ospedale Civile Sant'Andrea La Spezia*; Paolo Massirio, Luca Ramenghi *IRCCS Giannina Gaslini*

Lombardy Region

Debora Balestrieri *Ospedale di Cittiglio;* Federica Baltaro *Ospedale Niguarda di Milano;* Pietro Barbacini, Elisabetta Venegoni *Ospedale di Magenta;* Michele Barbato *Ospedale di Melegnano;* Lorena Barbetti *Ospedale di Esine;* Paolo Beretta *Ospedale di Como;* Bruno Bersellini *Ospedale di Sondrio;* Stefano Bianchi *Ospedale San Giuseppe di Milano;* Antonia Botrugno *Ospedale di Casalmaggiore;* Donatella Bresciani *Ospedale di Desenzano;* Alessandro Bulfoni *Pio X Humanitas di Milano;* Carlo Bulgheroni *Ospedale di Gallarate;* Orlando Caruso, Elena Pinton *Ospedale di Chiari;* Massimo Ciammella *Ospedale di Seriate;* Elena Crestani, Giulia Pellizzari *Ospedale di Pieve di Coriano;* Antonella Cromi *Ospedale di Varese;* Serena Dalzero, Nikita Alfieri *Ospedale San Paolo di Milano;* Rosa Di Lauro, Carla Foppoli *Ospedale di Sondalo;* Patrizia D'Oria, *Ospedale di Alzano;* Santina Ermito *Ospedale di Piario;* Massimo Ferdico *Ospedale di Vimercate;* Maria Fogliani, Guido Stevanazzi *Ospedale di Legnano-Cuggiono;* Roberto Fogliani *Ospedale di Sesto San Giovanni;* Ambrogio Frigerio *Ospedale di Rho;* Eleonora Fumagalli *Ospedale Macedonio Melloni ASST FBF-Sacco di Milano;* Roberto Garbelli *Brescia Istituto Clinico S. Anna;* Daniela Gatti *Ospedale di Manerbio;* Giampaolo Grisolia, Serena Varalta *Ospedale di Mantova;* Paolo Guarnerio *Ospedale San Carlo di Milano;* Enrico Iurlaro, Marta Tondo *IRCCS Cà Granda Ospedale Maggiore Policlinico-Mangiagalli Milano;* Stefano Landi *Ospedale di Gravedona;* Mario Leonardi *Ospedale di Iseo;* Stefania Livio, Chiara Tasca *Ospedale Buzzi ASST FBF-Sacco di Milano;* Anna Locatelli *Ospedale di Carate;* Giuseppe Losa *Ospedale di Melzo;* Massimo Lovotti *Como Valduce;* Anna Minelli *Ospedale di Gavardo;* Luisa Muggiasca *Ospedale di Garbagnate;* Giuseppe Nucera *Ospedale di Busto Arsizio;* Alessandra Ornati *Ospedale di Vigevano;* Luisa Patanè *ASST Papa Giovanni XXIII Bergamo;* Antonio Pellegrino *Ospedale di Lecco;* Francesca Perotti, Arsenio Spinillo *Fondazione IRCCS Policlinico San Matteo di Pavia;* Armando Pintucci *Ospedale di Desio;* Ezio Pozzi *Ospedale di Broni Stradella- Ospedale di Voghera,* Federico Prefumo *Spedali Civili di Brescia;* Anna Catalano *Brescia Fondazione Poliambulanza;* Aldo Riccardi *Ospedale di*

Cremona; Alessia Chiesa *Ospedale di Ponte San Pietro;* Tazio Sacconi *Ospedale di Asola;* Valeria Savasi, Silvia Corti *Ospedale Sacco di Milano;* Ubaldo Seghezzi *Ospedale di Saronno;* Vincenzo Siliprandi *Ospedale di Crema;* Marco Soligo, Beatrice Negri *Ospedale di Lodi;* Paolo Valsecchi *Ospedale San Raffaele;* Laura Vassena *Ospedale di Merate;* Federica Brunetti, Patrizia Vergani *Fondazione MBBM Ospedale San Gerardo Monza;* Antonella Villa *Ospedale di Treviglio;* Matteo Zanfrà *Ospedale di Tradate;* Alberto Zanini *Ospedale di Erba*

Autonomous province of Trento

Pietro Dal Rì *UO Rovereto*; Roberto Luzietti *UO Cles*; Fabrizio Taddei *UO Trento*; Fabrizia Tenaglia *UO Cavalese*

Emilia-Romagna Region

Lorenzo Aguzzoli, Alice Ferretti *Ospedale S.M. Nuova Reggio Emilia;* Patrizio Antonazzo, Lucrezia Pignatti *Ospedale Bufalini Cesena;* Angela Bandini, Isabella Strada *Ospedale G.B .Morgagni -L. Pierantoni Forlì;* Chiara Belosi *Ospedale degli Infermi Faenza;* Renza Bonini, Maria Cristina Ottoboni *Ospedale Guglielmo Da Saliceto Piacenza;* Fabrizio Corazza, Paola Pennacchioni *Ospedale Ss. Annunziata Cento;* Fabio Facchinetti, Giliana Ternelli *Azienda Ospedaliero-Universitaria Modena*; Alessandro Ferrari, Cristina Pizzi, *Ospedale S.M. Bianca Mirandola;* Tiziana Frusca, Stefania Fieni *Azienda Ospedaliero-Universitaria Parma;* Maria Cristina Galassi, Federica Richieri, *Nuovo Ospedale Civile Di Sassuolo S.P.A.;* Francesco Giambelli, Carlotta Matteucci *Ospedale S.M. Delle Croci Ravenna;* Pantaleo Greco, Danila Morano *Azienda Ospedaliero-Universitaria Ferrara;* Marinella Lenzi, Ilaria Cataneo, *Ospedale Maggiore Bologna;* Gialuigi Pilu, Marisa Bisulli, *Azienda Ospedaliero-Universitaria Bologna;* Maria Cristina Selleri *Ospedale di Bentivoglio;* Federico Spelzini, Elena De Ambrosi, *Ospedale Infermi Rimini;* Paolo Venturini, Francesca Tassinati *Ospedale B. Ramazzini Carpi;* Stefano Zucchini, Barbara Paccaloni, *Ospedale S.M. della Scaletta Imola*

Tuscany Region

Andrea Antonelli, Carlotta Boni *Ospedale Civile Cecina;* Maria Paola Belluomini, *S. Francesco Barga - PO Valle del Serchio* e *Generale Provinciale Lucca - PO San Luca,* Rosalia Bonura, *S. Maria della Gruccia - Ospedale del Valdarno,* Stefano Braccini, *SS. Cosimo e Damiano Pescia - Osp della Valdinievole,* Giacomo Bruscoli e Pasquale Mario Florio, *Nuovo Ospedale San Jacopo di Pistoia,* Giovanna Casilla, *SS. Giacomo e Cristoforo Massa - PO Zona Apuana,* Anna Franca Cavaliere, *Ospedale Santo Stefano Prato,* Marco Cencini, *Ospedali Riuniti della Val di Chiana,* Venere Coppola e Laura Migliavacca, *Ospedale Misericordia Grosseto,* Barbara De Santi, *PO Felice Lotti Pontedera,* Paola Del Carlo, *Ospedale S.Giovanni Di Dio Torregalli,* Carlo Dettori, *Nuovo Ospedale di Borgo S.Lorenzo,* Mariarosaria Di Tommaso e Serena Simeone, *Careggi - CTO Firenze - AOU,* Giuseppe Eremita, *Civile Elbano Portoferraio,* Sara Failli, *Ospedale Area Aretina Nord Arezzo,* Paolo Gacci, *S.M. Annunziata Bagno a Ripoli - Osp Fiorentino Sud Est,* Alessandra Meucci, *Le Scotte Siena - Azienda ospedaliera universitaria,* Filippo Ninni, *Riuniti Livorno,* Barbara Quirici, *Ospedale Unico Versilia,* Alessia Sacchi, *Ospedale dell'Alta Val d'Elsa Poggibonsi,* Cristina Salvestroni, *Ospedale S. Giuseppe Empoli,* Sara Zullino, *Ospedali Pisani Pisa - Az universitaria*

Campania Region

Annalisa Agangi *Ospedale Evangelico Villa Betania*; Salvatore Ercolano *P.O. "S. Leonardo" di Castellammare di Stabia*, Luigi Cobellis, Annunziata Mastrogiacomo *Ospedale di Caserta*; Maria Vittoria Locci *AOU Federico II Napoli*
