# Supplementary material for: Placental Characteristics of a Large Italian Cohort of SARS-CoV-2-Positive Pregnant Women
Source: Microorganisms. 2022 Jul 15;10(7):1435. doi: 10.3390/microorganisms10071435 (PMC9317507; doi:10.3390/microorganisms10071435)
Supplement: Supplementary file 1 [file microorganisms-10-01435-s001.zip › Supplementary File S3.pdf]

Supplementary File S3. The online data collection form (Italian)

Sezione A: INFORMAZIONI GENERALI

Codice identificativo donna:

A1. Cognome e nome della donna

A2. Data di nascita

A3. Data diagnosi di infezione da SARS-CoV-2 confermata

A4. Età gestazionale in settimane al momento della diagnosi di infezione da SARS-CoV-2

A5. PATOLOGIE PREGRESSE

Sì No

Asma che richiede steroidi orali o per via inalatoria

Malattie polmonari croniche

Malattie cardiovascolari croniche

Ipertensione cronica

AIDS/HIV

Obesità (BMI>30)

Diabete

A6. Specificare altre patologie pregresse

Sezione B: Esami diagnostici eseguiti durante il ricovero:

B1. RX del torace

Sì

No

B2. Ecografia del polmone

Sì

No

B3. TAC del torace

Sì

No

B4. Il referto ha evidenziato una polmonite interstiziale?

Sì

No

B5. Polmonite interstiziale dovuta a infezione da SARS-CoV-2

Sì

No

B6. Polmonite interstiziale dovuta a infezione da altri patogeni respiratori

Sì

No

B7. Specificare patogeni identificati come responsabili della polmonite

Sezione C: TERAPIA DURANTE IL RICOVERO

C1. Data e ora del parto

C2. Modalità del parto

Vaginale

Vaginale operativo (forcipe, ventosa)

TC in emergenza

TC in urgenza

TC programmato

C3.
Sì No
Ossigeno terapia
Ventilazione assistita
Ventilazione in assistenza con casco
Intubazione
Ricovero in Terapia sub-intensiva
Ricovero in Terapia intensiva
C4. Sono state rilevate condizioni di grave morbosità materna?
Sì
No
C5. Specificare:
Shock
Scompenso emodinamico
ARDS
Insufficienza renale
Altro, specificare
Altro, specificare
C6. La donna è deceduta?
Sì
No
Sezione D: NEONATO
DATI NEONATO 2
D1. La gravidanza è gemellare?
Sì
No
D2. Peso alla nascita in g
D3. Nato morto
Sì
No
D4. Punteggio di APGAR a 5 min
D5. Il neonato è deceduto?
Sì
No
D6. Peso alla nascita in g
D7. Nato morto
Sì
No
D8. Punteggio di APGAR a 5 min
D9. Il neonato è deceduto?
Sì
No
Sezione E: PLACENTA
E1. Modalità di invio della placenta
Fresca
Sottovuoto
Fissata

E2. Placenta pervenuta:
Intera
Frammentata
Lacerata
Sezione F: DESCRIZIONE MACROSCOPICA
F1. 1. Placenta
Singola
Gemellare fusa
Gemellare separata
F2. 1.1. Centile del peso della placenta:
F3. 2. Funicolo annesso
Sì
No
F4. 2.1. Se Sì, specificare:
Lunghezza in cm
Diametro massimo in cm
Diametro minimo in cm
F5. Inserzione
Centrale
Marginale
Velamentosa
F6.
numero di vasi al taglio
distanza dal margine placentare in cm
lunghezza del tratto vascolare intramembranoso in cm
F7. Spiralizzazione
Normospiralizato
Ipospiralizzazione
Iperspiralizzazione
F8. Presenza di: ***(tutte le risposte sono possibili)***
Costrizioni
Nodi
Torsioni
F9. Anomalie del funicolo
Sì
No
F10. Volume delle lesioni identificate in cm
F11.
Colorito delle lesioni identificate (biancastro/giallo verdastro)
F12. 3. Le membrano sono:
Complete
Incomplete
Sottili
Ispessite

F13. Lucentezza:
Francamente opaca
Francamente lucente
Altro, specificare
Altro, specificare
F14. Colore:
Biancastro
Verdastro
Giallastro
Brunastro
F15. Il punto di rottura è:
Evidente
Non evidente
F16. Distanza dal margine placentare in cm
F17. 4. Disco coriale (dopo rimozione delle membrane libere e del funicolo ombelicale)
Peso a fresco
Spessore in cm
Spessore massimo in cm
Spessore minimo in cm
N. di placche fibrose del versante fetale
N. di placche fibrinose del versante fetale
F18. 5. I vasi coriali:
A distribuzione magistrale
A distribuzione dispersa
F19. Presentano:
Aspetto normale
Congestione
Trombosi
Altro, specificare
Altro, specificare
F20. 6. Il parenchima placentare presenta: (tutte le risposte sono possibili)
Lesioni biancastre
Lesioni brunastre
Strie sfumate
F21. Se presentì le lesioni presentano:
Spessore in cm
F22. Localizzazione:
Marginali
Centrali
Subcorioniche
Intraparenchimali
Deciduali
Altro, specificare
Altro, specificare

F23. Se presentì le lesioni occupano:
% del volume placentare
F24. 7. I cotiledoni del versante materno sono:
Appiattiti
Prominenti
F25. 8. Numero di inclusioni eseguite:
Numero di sezioni di funicolo esaminate
Numero di campionamenti di membrane esaminati
Numero di campionamenti di parenchima esaminati
F26. Ulteriori inclusioni esaminate: (tutte le risposte sono possibili)
Sezione verso il versante fetale del funicolo
Punto di rottura
Membrane con inclusa la decidua capsulare
Full-thickness inserzione del funicolo longitudinale alla vena ombelicale
Sezione a cm 5 da inserzione del funicolo
Periferiche
Sulle lesioni identificate (una inclusione per ciascuna) con parenchima adiacente
Sezione G: DESCRIZIONE MICROSCOPICA
G1. Esame microscopico
Placenta gemellare corionicità:
Dicorionica diamniotica separata
Dicorionica diamniotica fusa
Monocorionica diamniotica
Monocorionica monoamniotica
Sezione H: 1. Membrane
H1. Amnios:
Assente
Cubico
Cilindrico
Esfoliato
Nodoso
Con metaplasia squamosa
Pigmenti
H2. Caratteristiche del corion (tutte le risposte sono possibili)
Edema
Emorragie
Calcificazioni
Lamelle cheratiniche
Pseudocisti
H3. Flogosi, grado:
Grado1 (non severo)
Grado2 (severo)
H4. Flogosi, stadio:
Stadio I (subcorionionite o corionite)
Stadio II (corionamnionite acuta, estesa al corion fibroso e/o amnion)
Stadio III (corionamnionite necrotizzante)

H5. Flogosi. infiltrato infiammatorio:
Linfo-plasmocitario
Linfo-granulocitario
Linfo-istiocitario
Sezione I: 2. Cordone ombelicale
I1. Amnios:
Assente
Cubico
Cilindrico
Esfoliato
Nodoso
Con metaplasia squamosa
Pigmenti
I2. Numero vasi
3
2 (un'arteria ed una vena)
Altro, specificare
Altro, specificare
I3. Flogosi, grado:
Grado1 (non severo)
Grado2 (severo)
I4. Flogosi, stadio:
Stadio I (vasculite corionica o flebite ombelicale)
Stadio II (coinvolgimento della vena ed almeno 1 delle arterie)
Stadio III (funisite necrotizzante)
I5. Flogosi. infiltrato infiammatorio:
Linfo-plasmocitario
Linfo-granulocitario
Linfo-istiocitario
I6. Flogosi, altro (tutte le risposte sono possibili)
Degenerazione della gelatina di Wharton
Edema
Necrosi
Sezione J: 3. Disco coriale
J1. MATURITA' GLOBALE
Età presunta dei villi:
II trimestre
III trimestre
J2. Maturità:
Immatura
Coerente con l'età gestazionale
Ipermatura
Dismatura (ipercrescita con immaturità)

J3. PIATTO AMNIOCORIALE
Vasi rami coriali:
Ectasici
Sclerotici
Trombizzati
Ispessimenti sottointimali
Vasculite
J4. Flogosi, grado:
Grado1 (non severo)
Grado2 (severo)
J5. LESIONI INFARTUALI
Infarti ischemici cotiledonari:
Recenti
In organizzazione
Di vecchia data
J6. Infarti ischemici dei rami villari:
Rari e focali
Frequenti in multiple diramazioni
J7. Emorragie intervillari:
Recenti
In organizzazione
Organizzate
Pseudocistiche
J8. Emorragie retroplacentari:
Recenti
In organizzazione
Organizzate
Pseudocistiche
J9. VILLI STAMINALI
Stroma dei villi
Cellularità:
Normale
Accentuata
Diminuita
J10. Stroma dei villi
Edema:
Diffuso
Focale
J11. Stroma dei villi
Altro:
Fibrosi
Sclerosi

J12. Vasi dei villi staminali:
Ectasici
Sclerotici
Trombizzati
Vasculite
Necrosi ischemica villare da ostruzione di vasi dei rami amnio-coriali
Vasculopatia occlusiva da flogosi
J13. VILLI INTERMEDI
Villi avascolari
J14.
Assenti Presenti Eccessivi
Villi intermedi immaturi:
Villi intermedi proliferanti:
J15. Stroma dei villi
Cellularità:
Normale
Accentuata
Diminuita
J16. Stroma dei villi
Cellule di Hofbauer:
Normali
Aumentate
J17. Stroma dei villi
Edema:
Diffuso
Focale
J18. Stroma dei villi
Altro:
Fibrosi
Sclerosi
J19. Vasi dei villi intermedi:
Ectasici
Sclerotici
Trombizzati
Stenosi da ipertrofia della tonaca muscolare
Vasculite
Necrosi ischemica villare da ostruzione di vasi dei rami amnio-coriali
J20. VILLI TERMINALI
Villi avascolari
J21. Ramificazione:
Iporamificati
Normali
Iperramificati
Regolare
Irregolare

J22. Fibrina intervillosa:
Lieve
Media
Marcata
J23. Necrosi fibrinoide dei villi:
Lieve
Media
Marcata
J24. Flogosi:
Lieve
Media
Marcata
J25. Precipitati calcici:
Apicali
Intervillosi
Marginali
J26. Trofoblasto:
Invaginazione
Inclusioni trofoblastiche
J27. Citotrofoblasto:
Accennato
Prominente
J28. Sinciziotrofoblasto:
Accennato
Prominente
J29. Gemme sinciziali:
Presenti
Assenti
J30. Nodi sinciziali:
Presenti
Assenti
J31. Membrana basale:
Mineralizzazione
Ispessimento membrana basale
Membrane vasculo-sinciziali
J32. Stroma dei villi
Cellularità:
Normale
Accentuata
Diminuita
J33. Stroma dei villi
Edema:
Diffuso
Focale

J34. Stroma dei villi
Infiltrato infiammatorio:
Linfo-plasmocitario
Linfo-granulocitario
Linfo-istiocitario
J35. Capillari dei villi terminali:
senza alterazioni significative
ectasici
ispessimento della membrana basale
ipovascolarizzazione
ipercapillarizzazione
coriangiosi
Sezione K: 4. Decidua
K1.
Presente nel materiale esaminato
Assente nel materiale esaminato
K2. Decidualizzazione:
Normale
Ridotta
K3. Stria Fibrinosa:
Regolare
Assottigliata
Focalmente interrotta
K4. Trofoblasto Intermedio:
Presente
Assente
Dismorfico (c.d. trofoblasto intermedio esaurito)
In ammassi
Neoplastico
K5. Vasi Deciduali:
Normali
Scarsamente modificati
Aterosi
Trombosi
Emorragici
Sezione L: INFORMAZIONI
L1. Vi preghiamo di segnalare eventuali informazioni utili all'interpretazione dei dati nel seguente campo note
I dati della scheda sono stati inviati e salvati correttamente. Grazie della collaborazione.
Powered by TCPDF (www.tcpdf.org)
