# Supplementary material for: Placental Characteristics of a Large Italian Cohort of SARS-CoV-2-Positive Pregnant Women
Source: Microorganisms. 2022 Jul 15;10(7):1435. doi: 10.3390/microorganisms10071435 (PMC9317507; doi:10.3390/microorganisms10071435)
Supplement: Supplementary file 1 [file microorganisms-10-01435-s001.zip › Supplemetary File S2.pdf]

Supplementary File S2. The standardised protocol for the biological samples collection, storage, and transport procedures (Italian). INDICAZIONI PER LA RACCOLTA, CONSERVAZIONE E TRASFERIMENTO DEI CAMPIONI BIOLOGICI
STUDIO ITOSS SULL'INFEZIONE DA SARS-COV-2 IN GRAVIDANZA E PUERPERIO

Il presente documento descrive le modalità di raccolta, conservazione e invio dei fluidi e tessuti biologici da sottoporre ad analisi microbiologiche e anatomopatologiche.
Solo le regioni o i centri nascita che hanno adottato il protocollo allargato dello studio eseguiranno il prelievo dei campioni biologici.
I prelievi biologici materni potranno essere effettuati solo ed esclusivamente alle donne che hanno letto l'informativa e firmato il modulo di consenso; i prelievi biologici neonatali potranno essere effettuati solo ed esclusivamente ai neonati di genitori che hanno letto l'informativa e firmato il modulo di consenso.
NB: un parere rilasciato dal Comitato Etico ISS, dopo l'avvio dello studio, prevede che in tutte le occasioni nelle quali il contesto organizzativo e/o assistenziale non permetta di raccogliere un consenso scritto il medico possa raccogliere il consenso verbalmente impegnandosi a inviare all'indirizzo itoss.cov2@iss.it una email con oggetto "Consenso al trattamento dei dati " nella quale specificare che a causa dell'emergenza derivante dall'epidemia, la gestante e il padre del bambino sono stati informati sui contenuti dell'informativa privacy relativa al protocollo dello studio e hanno dato il loro consenso al trattamento dei dati che riguardano la donna e il neonato. La dichiarazione dovrà riportare la data e il numero di identificativo della donna arruolata.

INDICAZIONI PER LA RACCOLTA DEI CAMPIONI BIOLOGICI

1. **Le donne che vengono arruolate durante la gravidanza** sia per assistenza ambulatoriale che per ricovero ospedaliero per infezione COVID 19 dovranno essere sottoposte a quanto descritto al punto 1. **NB:** le donne arruolate in gravidanza, **al momento del parto** dovranno comunque eseguire i prelievi previsti **al punto 2**
2. **Le donne che vengono arruolate al momento di travaglio e parto** dovranno essere sottoposte a quanto previsto al punto 2
   NB: Se al momento del ricovero per travaglio/parto la donna dispone di un referto positivo del tampone nasofaringeo **non occorre** ripetere il test.
3. **Le donne che vengono arruolate in puerperio** dovranno essere sottoposte a quanto previsto al punto 3
   NB: Se al momento del ricovero in puerperio la donna dispone di un referto positivo del tampone nasofaringeo **non occorre** ripetere il test

1. <u>All'arruolamento in gravidanza</u>

- **N. 2 tamponi UTM-virale vaginale**, eseguiti all'arruolamento a qualsiasi epoca gestazionale inserendo l'apposito tampone in vagina profondamente a livello del fornice vaginale posteriore.
- **Siero materno** (2 ml di siero, circa 5 ml di sangue intero) prelevato all'arruolamento a qualsiasi epoca gestazionale e al parto da prelievo venoso periferico. Va trasferito in provetta da siero (gel acrilico), la provetta va invertita 5-6 volte e lasciata a temperatura ambiente per 30' poi centrifugata a 1500-2000 giri/min per 10'. Quindi il siero va trasferito in altra provetta con tappo a vite da congelare a – 80°C (in alternativa a -20°C).

2. <u>Al momento di travaglio e parto</u>

- **N. 2 tamponi UTM-virale vaginale** eseguiti a qualsiasi epoca gestazionale durante il travaglio di parto inserendo l'apposito tampone in vagina profondamente a livello del fornice vaginale posteriore.
- **N. 2 tamponi UTM-virale rettale** eseguiti a qualsiasi epoca gestazionale durante il travaglio di parto inserendo l'apposito tampone per via rettale.
- **N. 2 tamponi UTM-virale nasofaringeo** eseguiti a qualsiasi epoca gestazionale durante il travaglio di parto.
- **Siero materno** (2 ml di siero, circa 5 ml di sangue intero) mediante prelievo venoso periferico

- **Siero cordonale** (2 ml di siero, circa 5 ml di sangue intero) prelevato al parto dopo avere pulito il cordone con garza sterile e soluzione fisiologica per eliminare il sangue esterno. Va trasferito in provetta da siero (gel acrilico). La provetta va invertita 5-6 volte e lasciata a temperatura ambiente per 30′ poi centrifugata a 1500-2000 giri/min per 10′. Quindi il siero va trasferito in altra provetta con tappo a vite da congelare a – 80°C (in alternativa a -20°C).
- **N. 2 tamponi UTM-virale placentari** prelevati sterilmente al parto. A seguito di un'accurata detersione del lato membranoso fetale con garza sterile e soluzione fisiologica, sollevare la membrana fetale e inserire il tampone in obliquo per circa 2 cm senza superare il lato materno.
- **Biopsie placentari**: prelevare sterilmente 3-4 biopsie placentari dal lato membranoso fetale (un cono con base di circa 3 cm includendo le membrane). I prelievi vanno lavati in soluzione fisiologica sterile e inseriti in contenitore sterile con tappo a vite. Congelare a – 80° C (in alternativa a -20°C).
- **Placenta** per esame istopatologico: prelevare al parto il restante campione anatomopatologico a seguito di biopsie tissutali già descritte.
- **N. 2 tamponi UTM-virale nasofaringeo** del neonato da eseguire alla nascita
- **Latte materno**: raccogliere sterilmente 2-5 ml del primo latte dopo il colostro in contenitore con tappo a vite. Congelare a – 80° C (in alternativa a -20°C).

3. <u>Durante il puerperio</u>

- **N. 2 tamponi UTM-virale nasofaringeo materni**
- **Siero materno** (2 ml di siero, circa 5 ml di sangue intero) mediante prelievo venoso periferico
- **Latte materno**: nelle donne che allattano raccogliere sterilmente 2-5 ml di latte in contenitore con tappo a vite. Congelare a – 80° C (in alternativa a -20°C).

INDICAZIONI PER LA CONSERVAZIONE DEI CAMPIONI BIOLOGICI

Tutti i campioni, essendo potenzialmente infetti, una volta prelevati devono essere conservati in un triplo contenitore, di cui possibilmente il più esterno dovrebbe essere una scatola (es di polistirolo).
Il contenitore esterno deve essere identificato inequivocabilmente con la scritta ben chiara e leggibile "COVID 19" e ogni campione, come ogni modulo di accompagnamento, deve riportare in chiaro il codice identificativo attribuito alla donna dallo studio ItOSS e riportato nella scheda di raccolta dati.

- **Siero cordonale e materno biopsie placentari** (3/4 pezzi per placenta) **e latte materno** (2-5 ml) devono essere immediatamente congelati dopo il prelievo, in ultracongelatore a -80°C o se non disponibile in freezer a -20°C.
- **Placenta:** immergere la placenta + membrane + cordone in formalina in un contenitore da chiudere ermeticamente e conservare a temperatura ambiente.

INDICAZIONI PER L'INVIO DEI CAMPIONI BIOLOGICI

Tutti i presidi sanitari delle regioni partecipanti eseguiranno in sede i tamponi nasofaringei materni e neonatali. Quelli che eseguiranno gli altri esami in laboratori della stessa Regione concorderanno localmente le procedure per lo stoccaggio e invio dei campioni, quelli che non eseguiranno gli altri esame in Regione potranno inviare i campioni al **Laboratorio di Anatomia Patologica dell'Ospedale Sacco (Pad. 19) a Milano**, nel rispetto delle seguenti indicazioni operative:

- Siero cordonale e materno, biopsie placentari e latte materno potranno essere spediti subito dopo la raccolta e il congelamento (come indicato sopra) oppure conservati in ultracongelatore/freezer, per poi essere spediti con cadenza mensile
  L'invio dovrà comunque essere sempre effettuato in ghiaccio secco.
- Membrane, cordone ombelicale e la placenta rimanente dopo il prelievo delle biopsie placentari, conservati in formalina, possono essere inviati con cadenza mensile.
  L'invio potrà essere effettuato a temperatura ambiente.
